# Degradation of small leucine-rich repeat proteoglycans by matrix metalloprotease-13: identification of a new biglycan cleavage site

**DOI:** 10.1186/ar1873

**Published:** 2006-01-03

**Authors:** Jordi Monfort, Ginette Tardif, Pascal Reboul, François Mineau, Peter Roughley, Jean-Pierre Pelletier, Johanne Martel-Pelletier

**Affiliations:** 1 Osteoarthritis Research Unit, University of Montreal Hospital Centre, Notre-Dame Hospital, 1560 Sherbrooke Street East, Montreal, Quebec H2L 4M1, Canada; 2 Genetics Unit, Shriner's Hospital for Children, 1529 Cedar Avenue, Montreal, Quebec H3G 1A6, Canada

## Abstract

A major and early feature of cartilage degeneration is proteoglycan breakdown. Matrix metalloprotease (MMP)-13 plays an important role in cartilage degradation in osteoarthritis (OA). This MMP, in addition to initiating collagen fibre cleavage, acts on several proteoglycans. One of the proteoglycan families, termed small leucine-rich proteoglycans (SLRPs), was found to be involved in collagen fibril formation/interaction, with some members playing a role in the OA process. We investigated the ability of MMP-13 to cleave members of two classes of SLRPs: biglycan and decorin; and fibromodulin and lumican. SLRPs were isolated from human normal and OA cartilage using guanidinium chloride (4 mol/l) extraction. Digestion products were examined using Western blotting. The identities of the MMP-13 degradation products of biglycan and decorin (using specific substrates) were determined following electrophoresis and microsequencing. We found that the SLRPs studied were cleaved to differing extents by human MMP-13. Although only minimal cleavage of decorin and lumican was observed, cleavage of fibromodulin and biglycan was extensive, suggesting that both molecules are preferential substrates. In contrast to biglycan, decorin and lumican, which yielded a degradation pattern similar for both normal and OA cartilage, fibromodulin had a higher level of degradation with increased cartilage damage. Microsequencing revealed a novel major cleavage site (... G_177_/V_178_) for biglycan and a potential cleavage site for decorin upon exposure to MMP-13. We showed, for the first time, that MMP-13 can degrade members from two classes of the SLRP family, and identified the site at which biglycan is cleaved by MMP-13. MMP-13 induced SLRP degradation may represent an early critical event, which may in turn affect the collagen network by exposing the MMP-13 cleavage site in this macromolecule. Awareness of SLRP degradation products, especially those of biglycan and fibromodulin, may assist in early detection of OA cartilage degradation.

## Introduction

Osteoarthritis (OA) is the most common rheumatologic disease, with high incidence and morbidity. Even though the early pathophysiological process remains to be elucidated, one of the first alterations in OA cartilage is a decrease in proteoglycan content [1]. Proteoglycans form a large group that can be classified into five families according to the structural properties of their core protein [2]. One group, termed the small leucine-rich proteoglycans (SLRPs), possesses a central domain of characteristic repeats that participate in protein-protein interactions [3]. The SLRPs can be divided into four classes based on gene organization and amino acid sequence homologies [1]: class I includes decorin, biglycan and asporin; class II includes fibromodulin, lumican, keratocan, PRELP (proline arginine-rich end leucine-rich repeat protein) and osteoadherin; class III includes epiphycan, mimecan and opticin; and class IV includes chondroadherin and the recently identified nyctalopin [4].

Although an understanding of the functions of SLRPs is only now emerging, most of the members bind specifically to other extracellular matrix constituents and contribute to the structural framework of connective tissues [3]. Moreover, some were shown to interact with various collagen types, including collagen type II, and to influence collagen fibril formation and interaction. These include decorin [5], fibromodulin [6], asporin [7], lumican [8], PRELP [9] and chondroadherin [10]. Moreover, fibromodulin, asporin, biglycan, decorin and lumican were also suggested to play a role in the OA cartilage process [11-13].

Decorin was the first in this series of molecules to be structurally defined. It contains one glycosaminoglycan chain, often dermatan sulfate, which can adopt complex secondary structures and form specific interactions with matrix molecules [3]. The decorin level in cartilage is by far the most abundant of the SLRPs, and in humans its level increases with increasing age [14]. Its proposed major functions are the regulation of collagen fibrillogenesis and maintenance of tissue integrity by its binding with fibronectin and thrombospondin [15-17] The closely related family member biglycan, despite its 57% of homology with decorin [18], does not interact with collagen under all conditions. Biglycan interactions appear to be primarily with type VI collagen. Biglycan has been identified at the surface of cartilage and in the pericellular region. In OA cartilage, a higher concentration was reported in the deeper layers of the tissue [19].

Fibromodulin contains up to four keratan sulphate chains [5] and was originally described as a collagen-binding protein. It is able to influence collagen fibril formation and maintain a sustained interaction with the formed fibrils [20]. Lumican, which is present at a high level in the cornea [21], has a widespread distribution in connective tissues [5,22,23], including cartilage [24]. Lumican and fibromodulin have been shown to bind to the same site on the collagen fibril [20,25]. Lumican modulates collagen fibrillogenesis and enhances collagen fibril stability [26].

Synthesis of collagen in normal and pathological cartilage is slow. However, in OA the integrity of the collagen network is impaired. This could result from defective linking of the collagen fibrils by molecules such as the SLRPs, thus interfering with the network stability, preventing its repair and accelerating its degradation. Cleavage of the SLRPs may then precede major destruction of the collagen and contribute to this process [20]. Data in the literature show that members of the matrix metalloprotease (MMP) family are able to cleave some SLRPs. MT1-MMP can cleave human recombinant lumican [27]; MMP-2, MMP-3 and MMP-7 cleave human recombinant decorin [15]; and MMP-13 cleaves bovine fibromodulin when this molecule is bound to collagen [20]. Purified bovine fibromodulin cannot be cleaved by human MMP-13 [20]. It was also recently shown that truncated disintegrin-like and metalloprotease domain with thrombospondin type I motifs-4 (ADAMTS-4) can cleave the MMP-13 susceptible bond of fibromodulin [28]. However, MMP-2, MMP-8 and MMP-9 do not cleave fibromodulin [20].

Although various MMPs are present in human OA cartilage, MMP-13 was demonstrated to play a major role. This enzyme, in addition to cleaving native collagen and having a higher activity on type II collagen than MMP-1, also acts to degrade various extracellular macromolecules including proteoglycans [29]. However, limited studies have been done on its effect on the SLRPs. We therefore investigated the ability of human recombinant MMP-13 to cleave members of two classes of the SLRPs (class I decorin and biglycan, and class II fibromodulin and lumican), derived from normal and OA human cartilage differing in the severity of the disease process. The results show that MMP-13 can degrade all four SLRPs, with fibromodulin and biglycan being preferential substrates.

## Materials and methods

### Specimen selection

Normal human cartilage (femoral condyles and tibial plateaus) was obtained from individuals within 12 hours of death at time of autopsy (*n *= 3; mean age [± standard deviation] 52 ± 14 years). These individuals had no history of joint disease and died from causes unrelated to arthritic diseases, including cardiorespiratory arrest, cerebral haemorrhage and pulmonary embolism. The tissue was examined macroscopically and histologically to ensure that only normal tissue was used.

OA human cartilage (femoral condyles and tibial plateaus) was obtained from patients undergoing total knee arthroplasty (*n *= 9; mean age [± standard deviation] 76 ± 5 years). All patients were evaluated by a certified rheumatologist who used the American College of Rheumatology criteria for OA of the knee [30]. These specimens represented early, moderate, or severe OA, as defined by microscopic criteria [31-33]. The Clinical Research Ethics Committee of the University of Montreal Hospital Center approved the study protocol and the use of human tissues.

### Proteoglycan extraction

Proteoglycans were extracted with 4 mol/l guanidinium chloride [34,35]. Briefly, cartilage was finely diced to pieces and extracted with 4 mol/l guanidinium chloride (Invitrogen Inc., Carlsbad, CA, USA) in 0.1 mol/l sodium acetate (pH 6.0) containing protease inhibitors (leupeptin [10 μg/ml], pepstatin [10 μg/ml], aprotinin [10 μg/ml], 1,10-phenanthroline [10 μg/ml] and phenylmethanesulphonyl fluoride [100 μg/ml]; EMD Biosciences Inc., La Jolla, CA, USA) at 4°C with continuous stirring for 48 hours. The extract was then separated from the cartilage residue by filtration through glass wool, and then dialyzed for 48 hours against 50 mmol/l Tris buffer (pH 7.5). One might argue that because the inhibitors were removed during the dialysis the endogenous MMPs could have been activated. However, because 1,10-phenanthroline is a zinc chelator, the catalytic zinc would also be removed by the dialysis, and so the MMPs would remain inactive.

### Analysis of SLRP cleavage by MMP-13

MMP-13 proteolytic activity was analyzed on human normal (*n *= 3) and OA cartilage having different levels of fibrillation corresponding to the different stage of the disease process. These were named slightly (*n *= 3), moderately (*n *= 3) and severely (*n *= 3) fibrillated cartilage. Proteoglycan extracts were incubated for 0–16 hours with human recombinant (rh)MMP-13 (R&D Systems Inc., Minneapolis, MN, USA) activated with 0.5 mmol/l aminophenylmercuric acetate (APMA; Kodak Inc., Toronto, ON, Canada) in 50 mmol/l Tris-HCl (pH 7.5) containing 10 mmol/l CaCl_2 _and 0.05% Brij 35 (Sigma-Aldrich Canada Ltd., Oakville, ON, Canada) at an MMP-13/proteoglycan ratio of 1:50 (100 ng/5 μg). Glycosaminoglycan content was determined using the 1,2-dimethylmethylene blue (DMMB) method [36]. The reaction was stopped by the addition of EDTA (Sigma-Aldrich Canada Ltd.) at a final concentration of 15 mmol/l. The samples were treated with 25 mU chondroitinase ABC (#C-2905; Sigma-Aldrich Canada Ltd.)/100 μl proteoglycan extract overnight at 37°C. In addition, a control was performed with the moderately fibrillated cartilage in which no MMP-13 was added and samples were incubated for 16 hours. Data were identical to those with the nonincubated specimens (data not shown).

In order to investigate MMP-13 specificity, RS 110–2481 (a synthetic specific MMP-13 carboxylate inhibitor generously provided by C Myers [Roche Bioscience, Palo Alto, CA, USA]) [37], was used. The Ki (nmol/l) for MMP-1, MMP-2, MMP-3, MMP-8 and MMP-13 were 1:100, 32, 19, 18 and 0.08, respectively. Briefly, samples from moderately fibrillated cartilage extract were treated with rhMMP-13 and RS 110–2481 at 1 and 50 nmol/l for the indicated time, and samples processed for Western blotting.

### Western blotting

Proteoglycan solutions were mixed with a sample buffer (62.5 mmol/l Tris-HCl [pH 6.8], 2% w/v sodium dodecyl sulphate, 10% glycerol, 5% β-mercaptoethanol, and 0.05% bromophenol blue) and electrophoresed on 4–20% Ready-Gels (Bio-Rad Laboratories Ltd., Mississauga, ON, Canada). They were then transferred electrophoretically to nitrocellulose membranes (Bio-Rad Laboratories Ltd.) and processed for Western immunoblotting. Blots were blocked in 2% low fat dry milk in Tris-buffered saline containing 0.05% Tween 20 (Sigma-Aldrich Canada Ltd.). As described previously [11], rabbit polyclonal antibodies raised against synthetic peptides corresponding to the carboxyl-terminus of the SLRP core proteins were used as primary antibodies for the detection of biglycan (1/5,000 dilution), fibromodulin (1/10,000 dilution), lumican (1/5,000 dilution) and decorin (1/5,000 dilution). The second antibody was a horseradish peroxidase-conjugated goat anti-rabbit immunoglobulin (1/10,000 dilution; Pierce, Rockford, IL, USA). Detection was performed by chemiluminescence using the Super Signal^® ^ULTRA chemiluminescent substrate (Pierce), in accordance with the manufacturer's specifications.

### Sequencing of biglycan and decorin degradation products

Bovine recombinant biglycan (15 μg) and decorin (15 μg; Sigma-Aldrich Canada Ltd.) were incubated for 1 hour at 37°C with APMA-activated rhMMP-13 in 50 mmol/l Tris-HCl (pH 7.5), containing 10 nmol/l CaCl_2 _and 0.05% Brij 35. The reaction was stopped by the addition of EDTA at a final concentration of 15 mmol/l. Glycosaminoglycan chains were removed by incubation with 0.1 unit chondroitinase ABC (#C-3667; Sigma-Aldrich Canada Ltd.) for 8 hours at room temperature, followed by boiling for 5 minutes with the electrophoresis sample buffer. To remove Asn-linked oligosaccharides, N-glycanase (0.3 unit; Roche Diagnostics, Laval, QC, Canada) and sample buffer containing 1.2% Nonidet P-40 (Roche Diagnostics) were added to the solution, which was then incubated again for 12 hours at room temperature. Degradation products were separated in 4–20% polyacrylamide gels (Bio-Rad Laboratories Ltd.). After electrophoresis, the gels were soaked in CAPS transfer buffer (10 nmol/l 3-cyclohexylamino-1-propanesulfonic acid, 10% methanol; pH 11.0) for 15 minutes at 0.25 A. After washing, the proteins were transferred onto PVDF membranes (Millipore Corporation, Bedford, MA, USA), which were washed in de-ionized water, stained with 0.1% Coomassie Blue in 50% methanol for 5 minutes, and then de-stained in 50% methanol and 10% acetic acid for 5–7 minutes at room temperature. Finally, the membrane was rinsed in de-ionized water, air dried and stored at room temperature. Amino-terminal amino acid sequencing of the protein band was performed on a Procise Protein Sequencer model 492 (Applied Biosystems, Foster City, CA, USA).

## Results

The use of human cartilage extracts to analyze SLRP degradation allowed study of all four SLRPs in a single extract under identical conditions, and permitted SLRP degradation to be carried out in a physiologically relevant extract of matrix proteins.

### MMP-13 degrades biglycan and decorin

**Figure 1:**
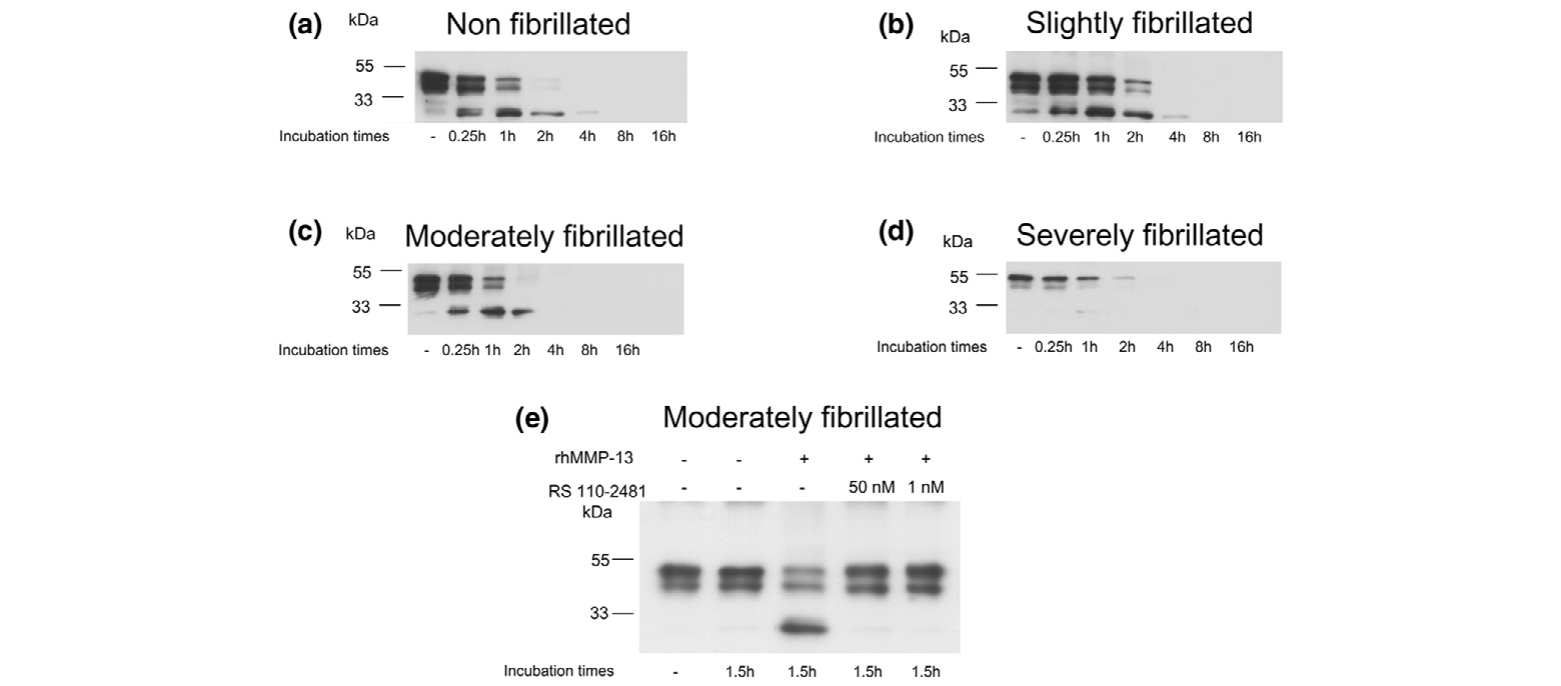
Representative Western blot of time course of MMP-13-induced degradation of biglycan. Human articular cartilage extracts were incubated with APMA-activated MMP-13 for the indicated times (0–16 hours). Panels are for extracts from **(a) **normal (nonfibrillated) cartilage and from **(b) **slightly, **(c) **moderately and **(d) **severely fibrillated OA cartilage. The bottom panel **(e) **relates to the extract from moderately fibrillated OA cartilage incubated for 1.5 hours with APMA-activated MMP-13 in the absence or presence of 50 or 1 nmol/l RS 110–2481 (a preferential MMP-13 inhibitor). APMA, aminophenylmercuric acetate; MMP, matrix metalloprotease; OA, osteoarthritis; rh, human recombinant.

Biglycan in human normal and OA cartilage migrated as a doublet at 48 and 45 kDa, representing intact and amino-terminally processed biglycan. MMP-13 degradation of biglycan was detected at 0.25 hours of incubation, and was almost complete at 2 hours (Figure 1). A fragment of about 28 kDa was generated. The biglycan profile from normal (nonfibrillated) to moderately fibrillated (Figure 1a–c) cartilage was similar whether the specimens were incubated in the presence or absence of MMP-13. Of note, in the specimens from nonfibrillated to moderately fibrillated cartilage not treated with MMP-13, a biglycan degradation product of a similar size to that generated by MMP-13 was already present, although in low amounts. Under MMP-13 treatment, there was an increase of the degradation product until complete digestion of the substrate. Interestingly, but not unexpectedly, in the severely fibrillated cartilage the biglycan was in low abundance (Figure 1d), which was possibly due to prior degradation and loss from the tissue. However, MMP-13 further cleaved the residual substrate.

To determine whether MMP-13 was the sole enzyme responsible for the cleavage, and not other enzymes present in the cartilage extracts, we further treated the samples from the moderately fibrillated cartilage with two concentrations (1 and 50 nmol/l) of a preferential inhibitor of MMP-13, namely RS 110–2481 [37]. Biglycan degradation was completely prevented at both concentrations tested (Figure 1e).

**Figure 2:**
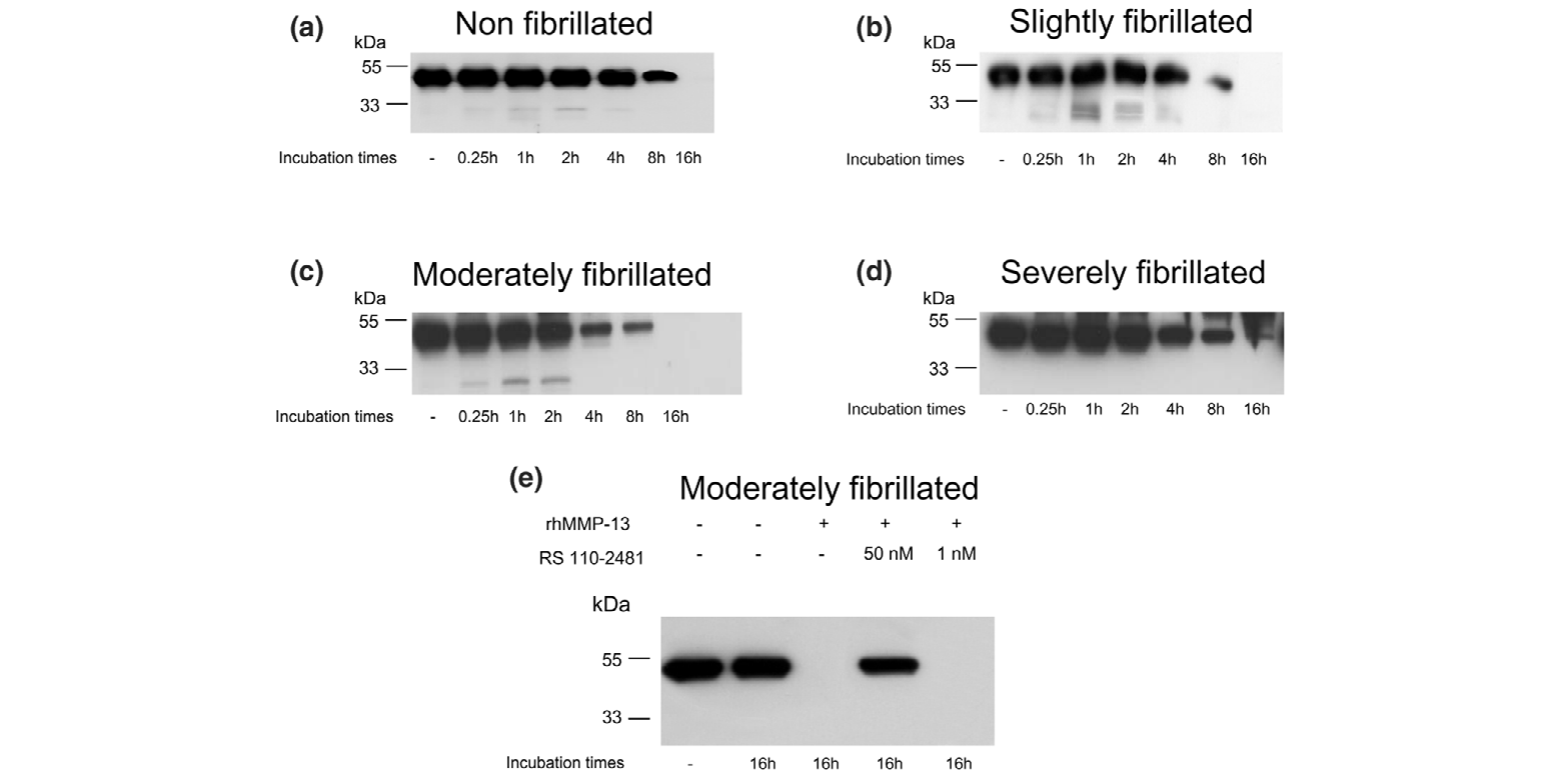
Representative Western blot of time course of MMP-13-induced degradation of decorin. Human articular cartilage extracts were incubated with APMA-activated MMP-13 for the indicated times (0–16 hours). Panels are for extracts from **(a) **normal (nonfibrillated) cartilage and from **(b) **slightly, **(c) **moderately and **(d) **severely fibrillated OA cartilage. The bottom panel **(e) **relates to the extract from moderately fibrillated OA cartilage incubated for 16 hours with APMA-activated MMP-13 in the absence or presence of 50 or 1 nmol/l RS 110–2481 (a preferential MMP-13 inhibitor). APMA, aminophenylmercuric acetate; MMP, matrix metalloprotease; OA, osteoarthritis; rh, human recombinant.

Decorin from normal and OA cartilage migrated as a single band of about 45 kDa. MMP-13 degradation of decorin was not detected until 4–8 hours of incubation, and proteolysis was complete by 16 hours (Figure 2). Two decorin fragments of about 30 and 28 kDa were detected. There was no major difference in the degradation pattern with the normal to moderately fibrillated cartilage (Figure 2a–c). In the severely fibrillated cartilage, no decorin fragment could be seen (Figure 2d). The ability of MMP-13 to degrade decorin was prevented in the presence of RS 110–2481 when the moderately fibrillated cartilage was incubated for 16 hours, but only at the higher concentration tested (50 nmol/l; Figure 2e). Of note, as decorin fragmentation was seen at early incubation time, this experiment was also performed at 1.5 hours and the data were identical (for instance, degradation was completely prevented at 50 nmol/l; data not shown).

### MMP-13 cleavage sites of biglycan and decorin

Amino acid sequencing analysis was performed with recombinant biglycan and decorin treated with MMP-13. In contrast to the Western blotting, which identifies carboxyl-terminal fragments, sequence analysis can identify the amino-terminus of all fragments.

**Figure 3:**
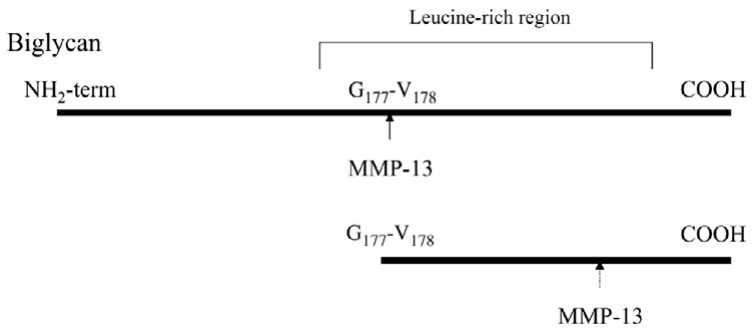
Biglycan cleavage sites generated by APMA-activated MMP-13. The arrow indicates the MMP-13 cleavage site, and the broken arrow the potential secondary MMP-13 cleavage site. APMA, aminophenylmercuric acetate; MMP, matrix metalloprotease; G, glycine; V, valine.

Sequence analysis of the biglycan fragments generated by MMP-13 treatment revealed a novel major fragment of 28 kDa. This fragment is generated by cleavage between positions 177 and 178 of the mature biglycan core protein, thus between glycine (G) and valine (V; Figure 3). A second biglycan fragment of 22 kDa was also identified by blotting and therefore possessed the carboxyl-terminal sequence. Presumably, this fragment is derived by cleavage within the 28 kDa fragment (Figure 3).

Sequence analysis of the two decorin cleavage fragments of 28 and 26 kDa showed that they possessed the same amino-terminus. The larger fragment is compatible with cleavage between positions 240 and 241 of the peptidic chain corresponding to a previously reported [15] cleavage site between the serine (S) and leucine (L). The exact cleavage site of the smaller fragment could not be identified.

The SLRP fragment sizes visualized on the gel used for sequencing were smaller than those observed on the gel used for Western blotting, possibly due to the treatment with N-glycanase in the former procedure. Of note, molecular weight determination by Western blotting is an approximation.

### Degradation of fibromodulin and lumican

**Figure 4:**
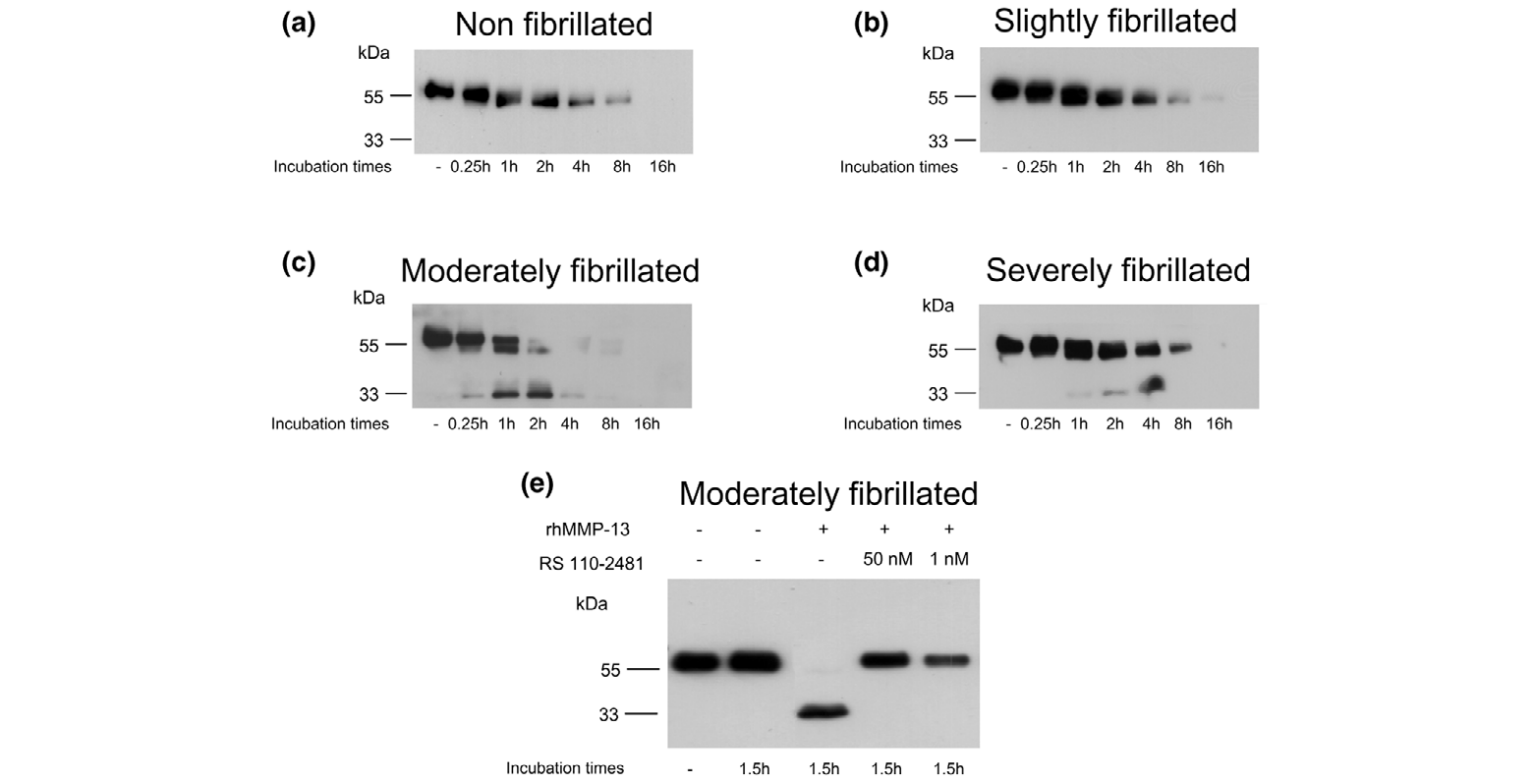
Time course of MMP-13 induced degradation of fibromodulin. Human articular cartilage extracts were incubated with APMA-activated MMP-13 for the indicated times (0–16 hours). Panels are for extracts from **(a) **normal (nonfibrillated) cartilage and from **(b) **slightly, **(c) **moderately and **(d) **severely fibrillated OA cartilage. The bottom panel **(e) **relates to the extract from moderately fibrillated OA cartilage incubated for 1.5 hours with APMA-activated MMP-13 in the absence or presence of 50 or 1 nmol/l RS 110–2481 (a preferential MMP-13 inhibitor). APMA, aminophenylmercuric acetate; MMP, matrix metalloprotease; OA, osteoarthritis; rh, human recombinant.

Fibromodulin from normal and OA cartilage migrated as a single component of about 60 kDa. MMP-13 induces fibromodulin degradation in a time-dependent manner, being detectable after 1–2 hours of incubation and complete by 16 hours (Figure 4). In the moderately and severely fibrillated cartilage, a degradation product of about 33 kDa was generated early under MMP-13 treatment (Figure 4c,d). The fragment initially increased in abundance with incubation time, and thereafter declined as the fibromodulin was further degraded. The specific MMP-13 inhibitor prevented fibromodulin degradation (Figure 4e).

**Figure 5:**
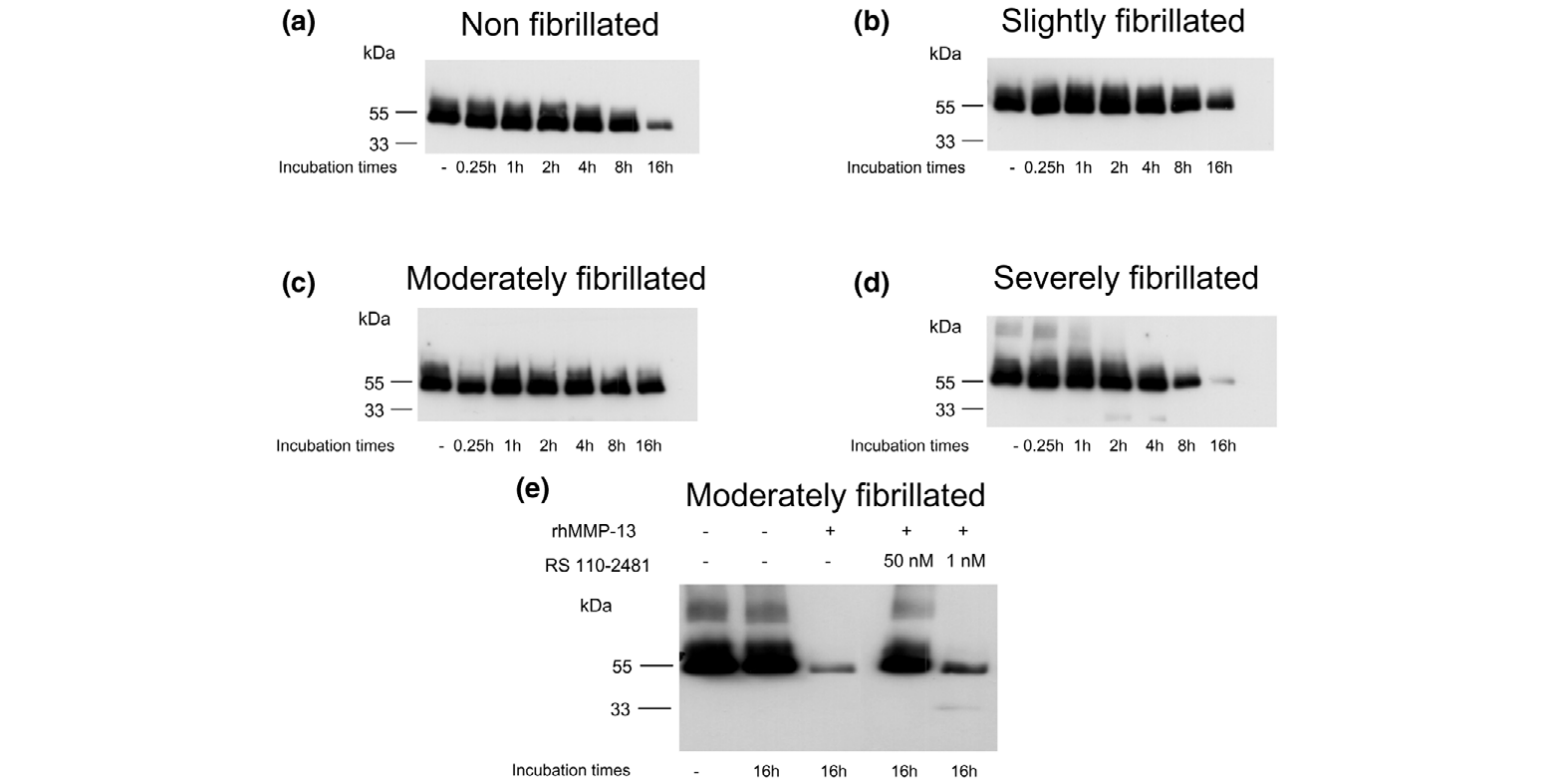
Time course of MMP-13 induced -degradation of lumican. Human articular cartilage extracts were incubated with APMA-activated MMP-13 for the indicated times (0–16 hours). Panels are for extracts from **(a) **normal (nonfibrillated) cartilage and from **(b) **slightly, **(c) **moderately and **(d) **severely fibrillated OA cartilage. The bottom panel **(e) **relates to the extract from moderately fibrillated OA cartilage incubated for 16 hours with APMA-activated MMP-13 in the absence or presence of 50 or 1 nmol/l RS 110–2481 (a preferential MMP-13 inhibitor). APMA, aminophenylmercuric acetate; MMP, matrix metalloprotease; OA, osteoarthritis; rh, human recombinant.

Lumican also migrated as a single component of 60 kDa. MMP-13-induced degradation was detected only after 8–16 hours of incubation (Figure 5). As for the other SLRPs, the specificity of MMP-13 was verified on extracts from moderately fibrillated OA cartilage, where lumican degradation was prevented by treatment with the MMP-13 specific inhibitor with a greater effect at 50 nmol/l (Figure 5e).

## Discussion

A major and early feature of cartilage degeneration is proteoglycan breakdown. MMP-13 has been shown to play an important role in OA cartilage degeneration by its effect not only on the collagen network but also on proteoglycans [2]. In the present study we investigated the ability of human MMP-13 to act on members of the SLRP proteoglycan family derived from human cartilage ranging from normal to advanced OA.

One emerging observation is that biglycan and fibromodulin are preferential substrates for MMP-13, whereas degradation of decorin and lumican is much less effective. This could imply that biglycan and fibromodulin are sensitive to both the gelatinolytic and collagenolytic activities of MMP-13, whereas decorin and lumican are more responsive to the gelatinolytic cleavage. Support for this hypothesis was provided by Imai and colleagues [15], who showed that decorin could be cleaved by MMP-2, MMP-3 and MMP-7, whereas cleavage with MMP-1 was negligible. The greater effect of MMP-13 than of MMP-1 on decorin could be due to the fact that the former enzyme has 44 times more gelatinolytic activity than does MMP-1 [38]. Moreover, and in agreement with this hypothesis, only 1 nmol/l of the inhibitor RS 110–2481 is sufficient to prevent collagenolytic activity, but 50 nmol/l is required to prevent gelatinolytic activity [37], and the effect of MMP-13 on biglycan and fibromodulin is abolished at both inhibitor concentrations whereas the effect on decorin and lumican is abolished only at the higher concentration.

Biglycan is found in the pericellular matrix of many connective tissues, and appears to play a role in regulating morphogenesis and differentiation [39]. Although biglycan is present in cartilage and is upregulated in the late stages of OA [13], its exact role in OA remains to be determined. The present data show that in some specimens a biglycan fragment of a similar size to that generated by MMP-13 is present in the cartilage as a minor component. It is possible that this *in situ *degradation product might not be cleaved at exactly the same site. This requires further study with an antibody recognizing the amino-terminal sequence of the fragment; however, such an antibody is not yet available. It is also possible that the biglycan degradation product may not be stably retained within the cartilage matrix and hence may not accumulate in large amounts. The study showed that the degree of biglycan degradation was independent of the extent of cartilage damage, although the amount of biglycan present in the severely fibrillated cartilage was significantly less than in normal to moderately fibrillated specimens. This suggests that, in the severely fibrillated specimens, biglycan has already been extensively degraded, leading to the loss of the epitope recognized by the antibody. Although we cannot exclude the possibility that proteases other than MMP-13 exerted an affect on this SLRP, this is unlikely because all endogenous carboxy, serine and MMPs should have been irreversibly inhibited by the inhibitor cocktail used in the extraction procedure. Although some cysteine proteases may survive the extraction procedure, it is unlikely that they remain active at pH 7.5, which was used for the incubation.

Our data also showed that MMP-13 induces two main biglycan fragments. The larger fragment possessed a new cleavage site (... G_177_-V_178_) in the leucine-rich region. The second smaller fragment possessed the same carboxyl-terminal sequence, indicating the presence of a second cleavage site. As the antibody used for immunodetection recognizes the carboxyl-terminal region of biglycan, cleavage at this second site must be after the G_177_-V_178 _cleavage site found in the larger fragment.

As mentioned above, Imai and colleagues [15] demonstrated the ability of three MMPs – namely MMP-2, MMP-3 and MMP-7 – to degrade decorin, and reported multiple cleavage sites. It seems likely that these MMPs cleaved within the leucine-rich region at different sites, because all fragments, albeit of different sizes, possessed the same amino-terminal sequence corresponding to that of the intact decorin core protein [15]. The present study revealed that MMP-13 degrades decorin into two fragments that also possess the same amino-terminal sequence as the intact decorin core protein. The products identified by amino acid sequencing from recombinant decorin were of 28 and 26 kDa. These may represent the amino-terminal fragments corresponding to the cartilage extract decorin fragments identified with a carboxyl-terminal antibody, because it appears that decorin cleavage occurs toward the centre of the molecule. One would expect the amino-terminal and carboxyl-terminal fragments to be of similar size. Because the degradation of decorin by MMP-13 appears to be due to its gelatinase activity rather than its collagenase activity, it is likely that one of the MMP-13 cleavages could be at the S_240_-L_241 _site, which is the cleavage used by gelatinase A (MMP-2) [15], and the other fragment would then be due to a cleavage amino-terminal of this site. This S_240_-L_241 _cleavage site is very plausible for MMP-13, because it is between aliphatic and hydrophobic amino acids, which are preferred by MMPs [40].

Interestingly, one of the characteristics of decorin is its interaction with active transforming growth factor (TGF)-β, thereby providing a tissue reservoir of this factor [41]. Our data showing MMP-13 cleavage in the leucine-rich repeats suggests the possibility that TGF-β may be released from the decorin after digestion with this MMP. We recently reported that, in OA cartilage, the TGF-β level is upregulated and responsible for the *in situ *increase in MMP-13 in this disease tissue [42,43]. The effect of MMP-13 on decorin, although not a preferential substrate, could be threefold. It may permit collagen degradation by its loss from the surface of the collagen fibrils; since data suggest that the leucine-rich repeats play a critical role in the interaction of SLRPs with collagens [44], it may result in loss of tissue integrity through the functional failure of decorin and biglycan interactions; and it may promote tissue degradation via TGF-β release, leading to increased MMP-13 production.

Lumican was reported to be present in human cartilage [24], but no direct evidence of its involvement in human OA has yet been reported. However, Young and colleagues [11] recently showed that lumican is upregulated in an ovine meniscectomy model of OA. This upregulated expression in degenerative cartilage was associated with increased lumican core protein deficient in keratan sulphate chains [11]. The present study showed that lumican degradation by MMP-13 occurs after an incubation period of 16 hours. This appeared independent of the level of fibrillation of the cartilage from which it was extracted, indicating that lumican degradation is independent of interactions with the various components in the different cartilage extracts.

Fibromodulin cleavage by MMP-13 has previously been demonstrated [20]. In human fibromodulin, cleavage occurs at the Y_63_-T_64 _site in the amino-terminal region of the molecule. In the present study MMP-13 degradation of fibromodulin generated a fragment of 30 kDa, which presumably corresponds to the fragment described by Heathfield and colleagues [20]. Of note, this fragment is generated in moderately and severely fibrillated cartilage, but not in normal or slightly fibrillated cartilage, reflecting an increased sensitivity of fibromodulin to degradation when the cartilage is more degenerated. This could be related to the presence of other components in the cartilage extracts that interact with the fibromodulin. Varying abundance of such components between the differently affected cartilages could then influence MMP-13 cleavage. The work by Heathfield and colleagues [20] suggests that cleavage of fibromodulin is dependent on its ability to bind type II collagen. There are two possibilities that could explain this situation. First, the ability of isolated SLRPs to interact with one another could result in the cleavage site being hidden. The recent description of decorin adopting a dimeric conformation in both the solution and crystal state may relate to this hypothesis, if other SLRPs behave in a similar manner [45]. It is possible that this dimeric conformation is removed when the SLRP binds to collagen and the MMP-13 cleavage site is then exposed. A second hypothesis could be that isolated SLRPs can act as zinc-binding proteins [46]. If this is a property of only free SLRPs, then in the absence of collagen or other binding partner the molecules could remove the zinc site necessary for MMP-13 function.

Although MMP-13 was shown to degrade type II collagen fibrils efficiently [47], it is possible that *in vivo *SLRP interaction may help to protect the fibrils by impeding access to the collagenase cleavage site. Data from this study are of importance in human OA pathophysiology, because MMP-13-induced SLRP degradation may represent an initial event in collagen fibril degradation, by exposing the collagen fibrils to proteolytic attack and permitting subsequent cartilage degeneration. *In vivo *identification of the SLRP degradation products, especially those of biglycan and fibromodulin, may assist in early detection of degeneration in OA cartilage.

## Conclusion

In this study we demonstrated the ability of human recombinant MMP-13 to cleave members of two classes of SLRPs (decorin, biglycan, fibromodulin and lumican) derived from normal and OA human cartilage differing in severity of the disease process. Although minimal cleavage of decorin and lumican was observed, cleavage of fibromodulin and biglycan was extensive, suggesting that both molecules are preferential substrates. We demonstrated that fibromodulin has a higher level of degradation with increased cartilage damage. We also characterized a novel major cleavage site for biglycan. We hypothesized that MMP-13-induced SLRP degradation may represent an early critical event in the process of cartilage degradation. Awareness of the SLRP degradation products may assist in early detection of OA cartilage degradation.

## Abbreviations

APMA = aminophenylmercuric acetate; MMP = matrix metalloprotease; OA = osteoarthritis; PRELP = proline arginine-rich end leucine-rich repeat protein; rh = human recombinant; SLRP = small leucine-rich proteoglycan; TGF = transforming growth factor.

## Competing interests

The authors declare that they have no competing interests.

## Authors' contributions

JM, GT, PR, PR, JPP and JMP contributed to the study design. JM, FM JMP acquired the data. JM, GT, PR, PR, JPP and JMP analyzed and interpreted the data. JM, PR and JMP prepared the manuscript. All authors read and approved the final manuscript.
